# Authors' reply

**Published:** 2010-02

**Authors:** M.V. Kalikar, V.R. Thawani, U.K. Varadpande, S.D. Sontakke, R.P. Singh, R.K. Khiyani

**Affiliations:** Department of Pharmacology, Government Medical College, Nagpur – 440 003, India; 1 Department of Preventive and Social Medicine, Government Medical College, Nagpur – 440 003, India; 2 Department of Skin and Venereal Diseases, Government Medical College, Nagpur – 440 003, India; 3 Department of Rasa Shastra, Government Ayurvedic College, Nagpur – 440 009, India

Sir,

We would like to thank Dr. Akhtar for his comments[1] on our paper.[2]

We agree that the baseline CD4 count of some participants (as factually reported by us) in TCE and placebo group was <200 / ul. This study was done in 2005-06 when free ART for HIV patients was not available anywhere in our city. Those patients coming to our hospital, who were not able to afford the ART from market and who volunteered to participate were included in this trial. Thus, even otherwise, the participants were just receiving symptomatic treatment for their opportunistic infection only. Had the ART treatment been available in our hospital at that time, we would have certainly put them on HAART and used TCE as add-on therapy.

Fall in Hb% cannot be attributed to TCE as it occurred in placebo group also at the end of six months and was comparable in both groups with no statistically significant difference [Table 4].

Kruskal Wallis test was used by us in consultation with qualified statistician (see our acknowledgement) and the explanation offered for using this test was that if one wants to compare the mean of two groups and one is not sure about the distribution or sample size is small, a non parametric test like Kruskal Wallis test is used.
